# Assessment of behaviour, performance, and blood parameters in Japanese quail under toxic stress of imidacloprid and its modulation by vitamin B complex

**DOI:** 10.1038/s41598-026-66200-1

**Published:** 2026-08-21

**Authors:** Doaa S. Abd El-Maguid, Sary K. Abd-elghaffar, Hagar Mohamed, Ramadan D. El Shoukary, Ayman S. M. Abdel-Maguid, Mahmoud Abdel-Nasser

**Affiliations:** 1 Department of Forensic Medicine and Toxicology, Faculty of Veterinary Medicine, New Valley University, New Valley, Egypt; 2 Department of Pathology and Clinical Pathology, School of Veterinery Medicine, Badr University in Assiut, Assiut, Egypt; 3 Department of Pathology, Faculty of Veterinary Medicine, Assiut, Egypt; 4 Department of Animal Wealth Development, Faculty of Veterinary Medicine, New Valley University, New Valley, Egypt; 5 Department of Agricultural Economics, Higher Institute for Agricultural Cooperation and Extension, in Assiut University, Assiut, Egypt; 6 Department of Forensic Medicine and Toxicology, Faculty of Veterinary Medicine, Assiut University, Assiut, Egypt

**Keywords:** Imidacloprid, Becozym, Feeding behaviors, Fear, Quail performance, Biochemistry, Environmental sciences, Physiology, Zoology

## Abstract

Pesticides are classified as a major source of contamination and a substantial risk to both humans and animals. The fourth generation of pesticides is the neonicoetinoid insecticides. Imidacloprid is a widely used neonicotinoid insecticide utilized in crop protection. The current study was conducted to assess behaviour, performance, and some blood parameters due to long-term use of sub-lethal doses of IM in Japanese quails and the protective impact of vitamin B complex. Non-sexed, one day-old chicks were split into three groups of 45 each. Oral administration of imidacloprid in water at dose of 1/10 of LD50 (4.43 mg/kg from 70%) (Group 1), 1/10 of IM LD50 + 5 ml/kg Vitamin B complex were given orally (Group 2), and the control was received only water (Group 3). Administration applied twice weekly for two weeks old age quails for 12 weeks. Data on behavior, performance, and welfare were collected weekly (weeks 1, 2, and 3), while blood samples were taken biweekly (weeks 2 and 4) until the fourth week. Finally, pathological samples were taken exclusively at week 12 of the experiment. The obtained data revealed that, from a behavioral perspective, there was a significant decrease in feeding behaviors (feeding and drinking), comfort behavior, and locomotor activity (standing), alongside an increase in crouching behavior. Growth performance was also adversely affected, as evidenced by a significant decrease in final body weight and body weight gain, paired with a significant increase in the feed conversion ratio despite a decrease in total feed intake. In terms of welfare, a significant increase in tonic immobility time and its index was observed, both of which were partially ameliorated by the vitamin B complex supplementation. Finally, on the biochemical side, there was a significant increase in specific liver and kidney parameters (creatinine, ALT, and AST), as well as a significant decrease in total protein, albumin, and acetylcholinesterase (AChE). Histopathology of sciatic nerves showed different damages. Some of these alterations were partially attenuated by addition of vitamin b-complex as (Creatinine and total protein). It was concluded that, the long-term use of low dose imidacloprid resulted in deterioration in various behavioral, biochemical, and pathological measurements, affecting the body performance. The administration of vitamin B complex could ameliorate many of these hazardous effects.

**Supplementary Information:**

The online version contains supplementary material available at https://doi.org/10.1038/s41598-026-66200-1.

## Introduction

Ecotoxicological behaviour studies alterations in behavior resulting from exposure to toxic substances in order to clarify their potential impacts and other factors on people and societies^1^. It is the integration of an understanding of individuals’ accommodating responses to environmental stressors, markedly chemical pollutants, besides the consequent impressions of those behavioural alterations on the people and associations of the ecological system. Over the past 20 years, neonicotinoid pesticides have gained widespread use in agriculture, but their growing global application has been linked to detrimental impacts on avian populations^2^.

In agricultural settings, particularly in poultry farming, the health of broiler chickens is of paramount importance for both economic viability and animal welfare^3^. The growing concerns regarding the usage of pesticides such as IMI in agriculture have highlighted the need for solutions to mitigate their deleterious effects on poultry^4^.

Imidacloprid (IMI) (1- (6- chloro-3- pyridylmethyl) -N- nitroimidazolidin -2 ylideneamine), is the initial systemic insecticide among neonicotinoids structured of chloronicotinyl and it is chiefly hazardous to birds^5^. The fatal IMI dose 50% ranges from 13.9 to 283 mg/kg body weight, which is categorized as 'greatly to moderately severe’^6^. Effects of brief-term exposure to imidacloprid in birds are well-researched^7^, nevertheless, the prolonged exposure was needed to recognize late impacts at doses that arise in agroecology^6^.

Neonicotinoids’ neurotoxic sequels can emerge through diverse behavioural, physiological, and biochemical, besides pathological, alterations in either the central or marginal nervous networks. Imidacloprid, a popularly utilized neonicotinoid, also-ran a key core of studies due to its important position in present-day pest control and its prospect of harming the health of the environment and humans^8^. Nevertheless, overuse of this group can lead to sharp environmental issues besides health risks^9^.

Behavioral responses are referred to as “early admonition signals” because they mostly happen at lower doses of chemical exposure, than conventional final stages^10^. Moreover, Behavioral and body weight assessments act as early potential non-destructive field monitoring biomarkers of IMI effects^11,12^ and in this context, evaluating related specific behavioral traits, such as tonic immobility supplies a variety of behavioral indicators^12,13^.

Broilers’ exposure to IMI-contaminated feed induced significant adverse effects on growth performance^14^ diminished productivity and compromised health, which in turn can affect the agricultural industry’s sustainability^4^, and neurobehavioral abnormalities^15^.

The neurotoxicity arises from its role as an insect-nicotinic acetylcholine receptor (nAChR) inhibitor with affinity for mammalian nAChRs^16^ causing deficit sensorimotor functions, increased fear-related behavior, low exploratory behavior^17,18^ reduced mobility, decreased anti-predatory behavior and loss of body weight^6^.

Previous works has been done on cumulative neurobehavioral and neurotoxic effects of IMI and the neuroprotective potential of natural dietary addition such as Moringa, curcumin and selenium during a specific 6-week growth period of broiler chickens, as in Abd-Elhakim^19^ and^4^ and thymol in rats^16^.

We used the species of Coturnix japonica, as a model trend to assess the outcomes of long-standing exposure to an anticipate environment. It is a species considered for (eco) toxicological studies, an ideal animal model. Ever after, they exhibit ease in handling capture, impedance to illnesses and inexpensive maintenance payments^20^.

We proposed the theory that water intake carrying IMI motives an imbalance in behavior, performance, welfare and some blood parameters, and that vitamin B complex may help alleviate this toxicity. Our work continues from 14 to 42 days, still 28 days to study the weekly, biweekly and cumulative effects on behaviour, performance and tonic immobility tests as welfare tests. Besides that, some blood parameters were measured. In addition, a pathological histological examination is performed at the end of the experiment.

## Materials and methods

1. Chemicals used

Imidacloprid (IMI), (Joun 70% WG produced by Aspire Agro chemicals Company), vitamin B complex (Epicoenzym produced by Epico).

2. Bird and experimental design.

A total of 135 unsexed, seven-day-old Japanese quails (*Coturnix coturnix japonica*) were obtained from the Faculty of Agriculture, New Valley University. The experiment was conducted at the Animal House Unit of the Faculty of Veterinary Medicine, New Valley University. The birds were acclimated to laboratory conditions for 7 days prior to the start of the experimental period. At 14 days of age, the quails were randomly assigned to three experimental groups, with each group consisting of three replicates of 15 birds (45 birds per group).

The birds were reared in multi-tiered batteries, with each replicate allocated an area of approximately 40 × 50 × 30 cm, following the husbandry and management guidelines described by Abdelfattah et al.^21^. A lighting schedule of 23 h of light and 1 h of darkness was maintained throughout the study. The ambient temperature was initially set at 37 °C during the first week and was subsequently decreased at a rate of 3 °C per week. A mash diet containing 24% crude protein (CP) and 2.8 Mcal of metabolizable energy per kilogram (2.8 Mcal ME/kg) was utilized. Throughout the experimental period, the birds had ad libitum access to the diet and clean water.

The experimental groups were designated as follows:

- Control Group (C): Provided with standard potable drinking water without any additives.
- IMI Group: Administered a dose corresponding to 1/10 of the LD50% of imidacloprid (4.43 mg/kg body weight from a 70% formulation) via the drinking water.
- IMI + B-complex Group: Administered a combination of 1/10 of the LD50% of imidacloprid (4.43 mg/kg body weight) and 5 mL/kg of vitamin B complex syrup (Becozyme) via the drinking water.

Imidacloprid and the Vitamin B complex treatments were freshly prepared and administered twice a week for duration of 4 weeks.

## Study parameters

### Estimation of behavior

The behavioral analysis including all assessed behaviors was performed weekly by twice daily (morning and afternoon) for two non-consecutive days per week by direct observation scanning method^22^. All behavioral data were illustrated as the frequency of behavioral occurrences by the bird at each observation time. According to the ethological Table 1 according to Abd-Elhakim et al.^19^, behavior was taken at w1, w2, and w3 and not taken at w4 of the experiment due to sexual maturity change.

**Table 1:** Quail ethogram.

Ethological table	
*Ingestive behavior*	
Feeding:	The bird’s head is within the feeding trough apparently consuming feed
Drinking:	The bird was seen nibbling at the nipple drinker. Probably drinking water
*Locomotor behavior*	
	Takes at least two consecutive steps, excluding scratching in the litter with its feet
Standing:	The bird’s feet make contact with the ground, but no other body parts do
*Comfort behavior*	
Preening:	The bird manipulates its feathers through subtle pecks
Wing and leg stretching:	Open beak at a rate that is faster than usual
Ruffling and shaking of feathers:	The bird ruffles all of its feathers and shakes itself
Crouching:	The ventral section of the body is in contact with the ground, with a bent knee and closed eyes

### Estimation of welfare (emotional reactivity)

The welfare analysis as tonic immobility test which consider attest for a bird’s level of fear was performed weekly^23^. The bird was placed on its right side because the RLP was an almost efficient strategy to prompt TI in chickens on the floor^24^ and held there for 10 s. Before the birds stood alone, the time that had passed was measured. After releasing the bird, if it did not stay still for at least 10 s, the tonic immobility attempt was deemed to have unsuccessful and a new attempt was made. The number of times tonic immobility was tried was recorded. w1, w2, w3 and not taken at w4 of the experiment due to sexual maturity change.

During the test, two factors have been tracked: the total number of inducement trials besides the length of tonic immobility duration. Quails with a few numbers of induction attempts and a prolonged period of tonic stagnation were expressed as being the highly fearful. A ternary parameter measured following the test was the tonic immobility index, which assigns greater weight to tonic immobility that is induced with ease and lesser weight to tonic immobility that necessitates multiple inducements [index equals to (6-number of inducements) × duration of tonic immobility]^25^.

### Estimation of productive performance

Body weight, and feed intake were weighted weekly and the gaining weight and feeding conversion efficiency were measured at w1, w2, and w3 and not taken at w4 of the experiment due to sexual maturity change.

### Estimation of some blood parameters

Five birds randomly taken from each group every 2 weeks were weighed and humanely euthanized by halal slaughter according to Islamic procedures^26,27^ during drain of blood, around 3 cm of blood from each slaughtered quail was collected in a test vial without anticoagulant at these periods,after 2 weeks, 4 weeks. Blood samples without anticoagulant were centrifuged for ten minutes at three thousand rotations per minute after being left at ambient temperature for 15 min in order to get clean residual serum. The serum samples at − 80 °C were kept frozen until the analysis.

Total protein, Albumin, Creatinine, Acetylcholine esterase, Aminotransferase Aspartate (AST) and Alanine (ALT) were utilizing a spectrophotometer apparatus (T80 UV/VIS PG instrument Ltd, UK) using trading kits.

### Histopathological evaluation by transmission electron microscope

Sciatic nerves 1 × 2 mm were dissected from of each bird after 12 weeks of exposure as follows:

#### A procedure used to isolate the sciatic nerve

A longitudinal incision was done to separate the quail’s biceps femoris and tensor fasciae latae muscles. Evidence of the sciatic nerve was appearimg. By following this evidence and by deviating the biceps femoris and tensor fasciae latae muscles; a clear, white, cord-like form (cord alike) sciatic nerve was dissected longitudinally from its emerging point; foramen ischiadicum to the point it exited^28^.

The dissecated nerve was fixed in 5% cold-buffered glutaraldehyde instantly after anatomizing the animals. The specimens were afterwards cleaned 3–4 times in cacodylate buffer (pH 7.2) for 20 min every time and afterwards fixed for 2 h in 1% osmium tetraoxide. After that, samples were washed for four times in the same buffer and dehydrated by developing grades of ethyl alcohol for 2 h (30, 50, 70, 90 and 100%) were done for each. Then, the samples were immersed in an Epon-Araldite mixture in accordance with the Electron Microscope Unit technique, Assiut University^29^. From the immersed blocks, semithin sections were prepared by LKB ultramicrotome in thicknesses of 0.5–1 μm to orient the tissue then taken a photo by SC30 Olympus camera. Afterwards, ultrathin sections of 500–700 Å thickness were created using up Leica AG ultramicrotome and stained with uranyl acetate and lead citrate and lead citrate, as usual and were examined with a transmission electron microscope 100 CXII electron microscope at 80 kv and photographed by a CCD digital camera model XR-41.

### Statistical analysis

All statistical analyses were performed using GraphPad Prism software (version 6.0). Before conducting parametric analyses, data normality was assessed using the Shapiro–Wilk test, and the homogeneity of variances was evaluated using Brown-Forsythe or Bartlett’s test. Group differences were subsequently determined via a one-way analysis of variance (ANOVA), followed by Tukey’s post hoc test for multiple comparisons. Data are expressed as mean values ± standard error of the mean (SEM). Differences were considered statistically significant at *p* < 0.05. On all corresponding charts, significance levels are indicated as follows: *p < 0.05, * *p < 0.01, and * * *p < 0.001.

## Results

### Effect of IMI exposure on Quail behavioral and welfare parameters

**Fig. 1:**
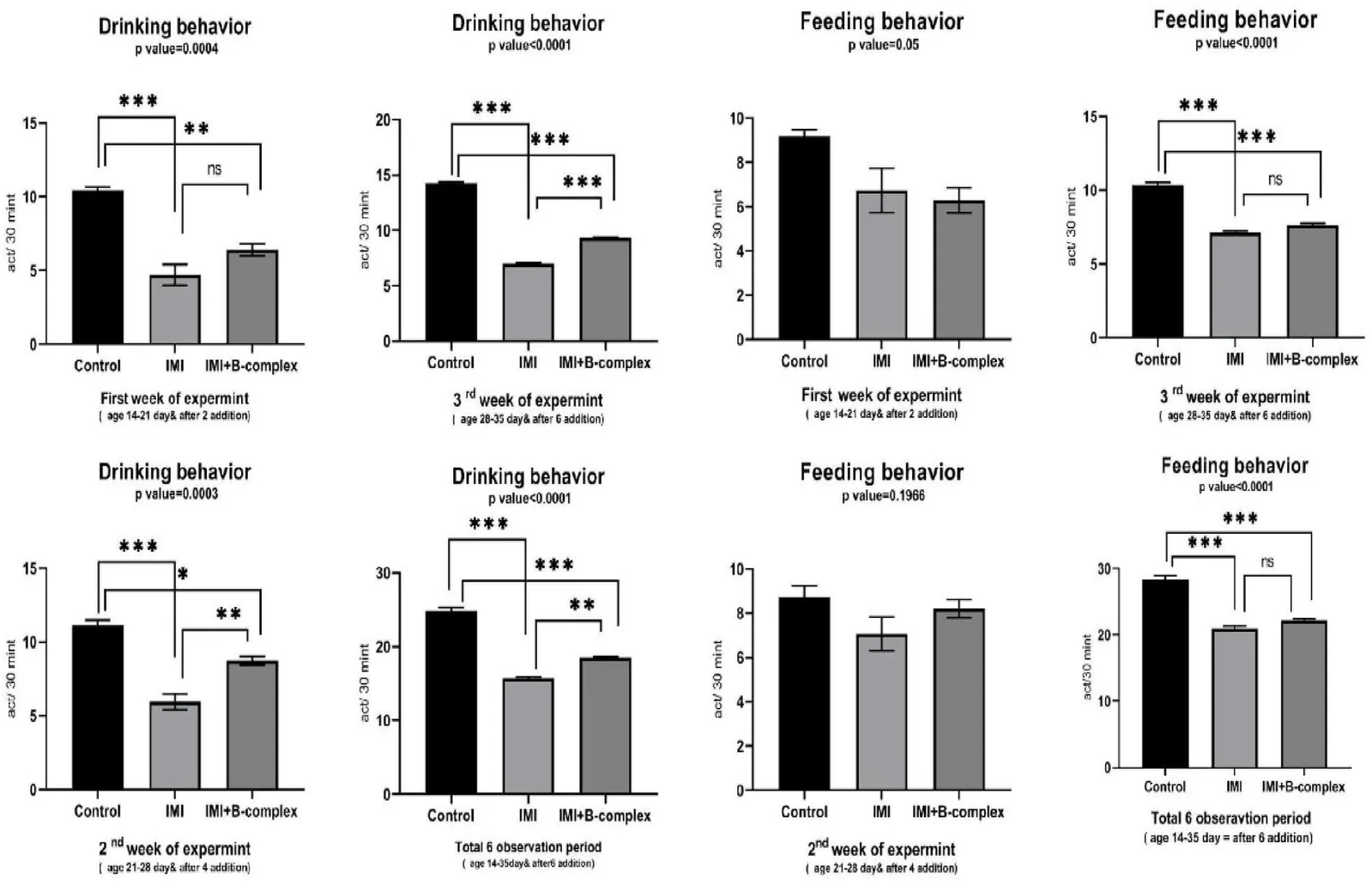
Effect of imidacloprid and vitamin b complex on quail ingestive behavior.

**Fig. 2:**
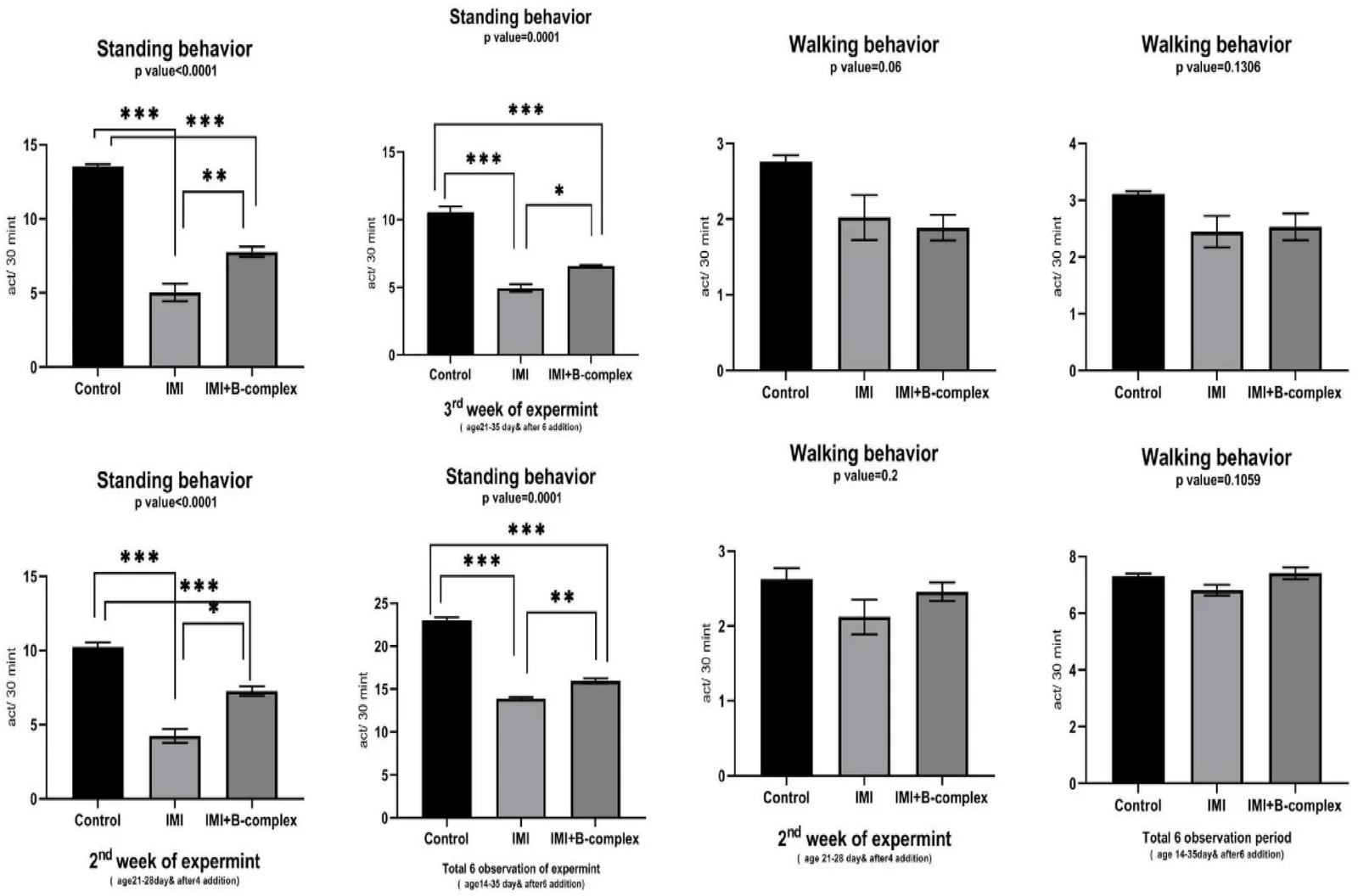
Effect of imidacloprid and vitamin b complex on quail locomotor behavior.

**Fig. 3:**
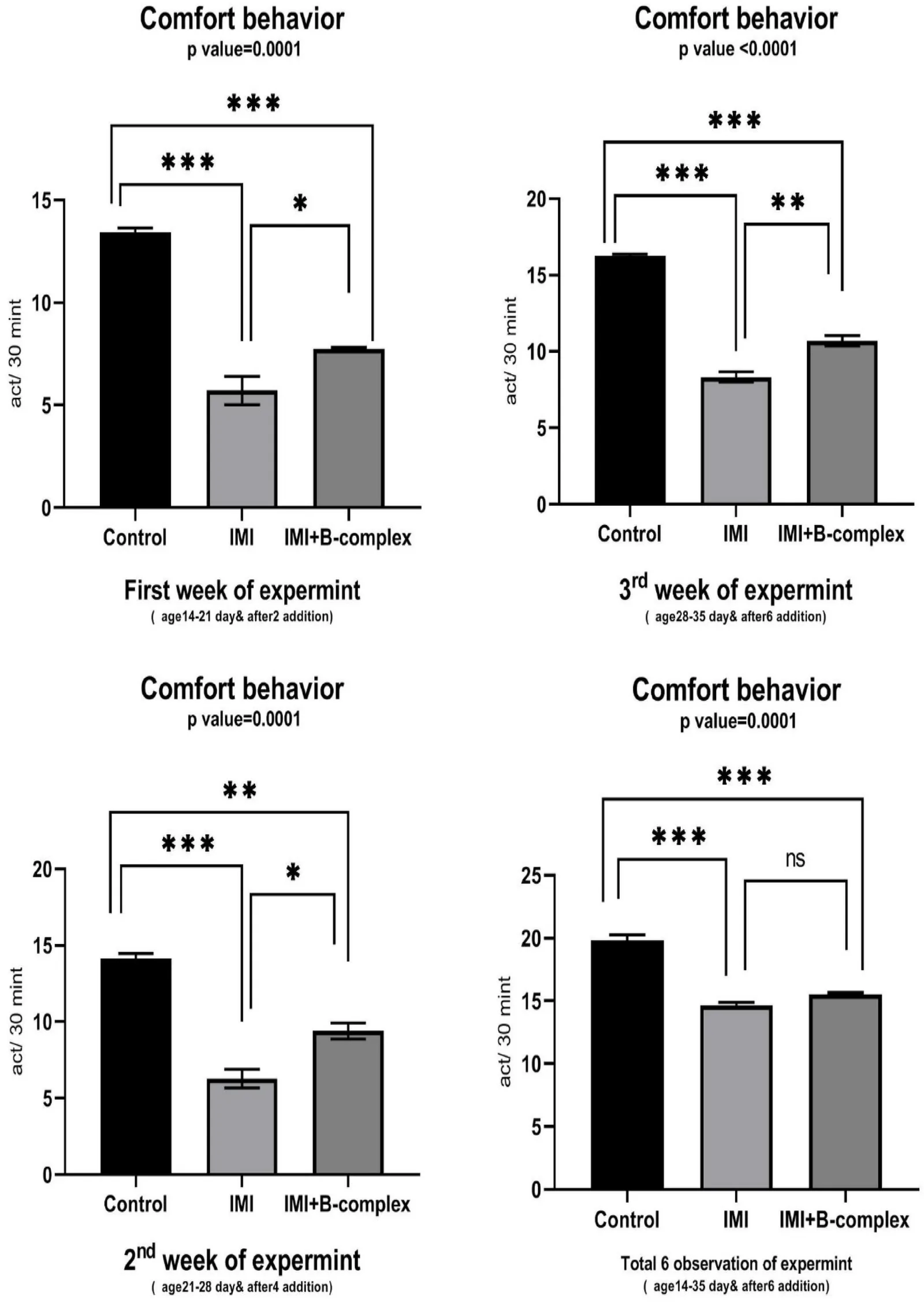
Effect of imidacloprid and vitamin b complex on quail comfort behavior.

**Fig. 4:**
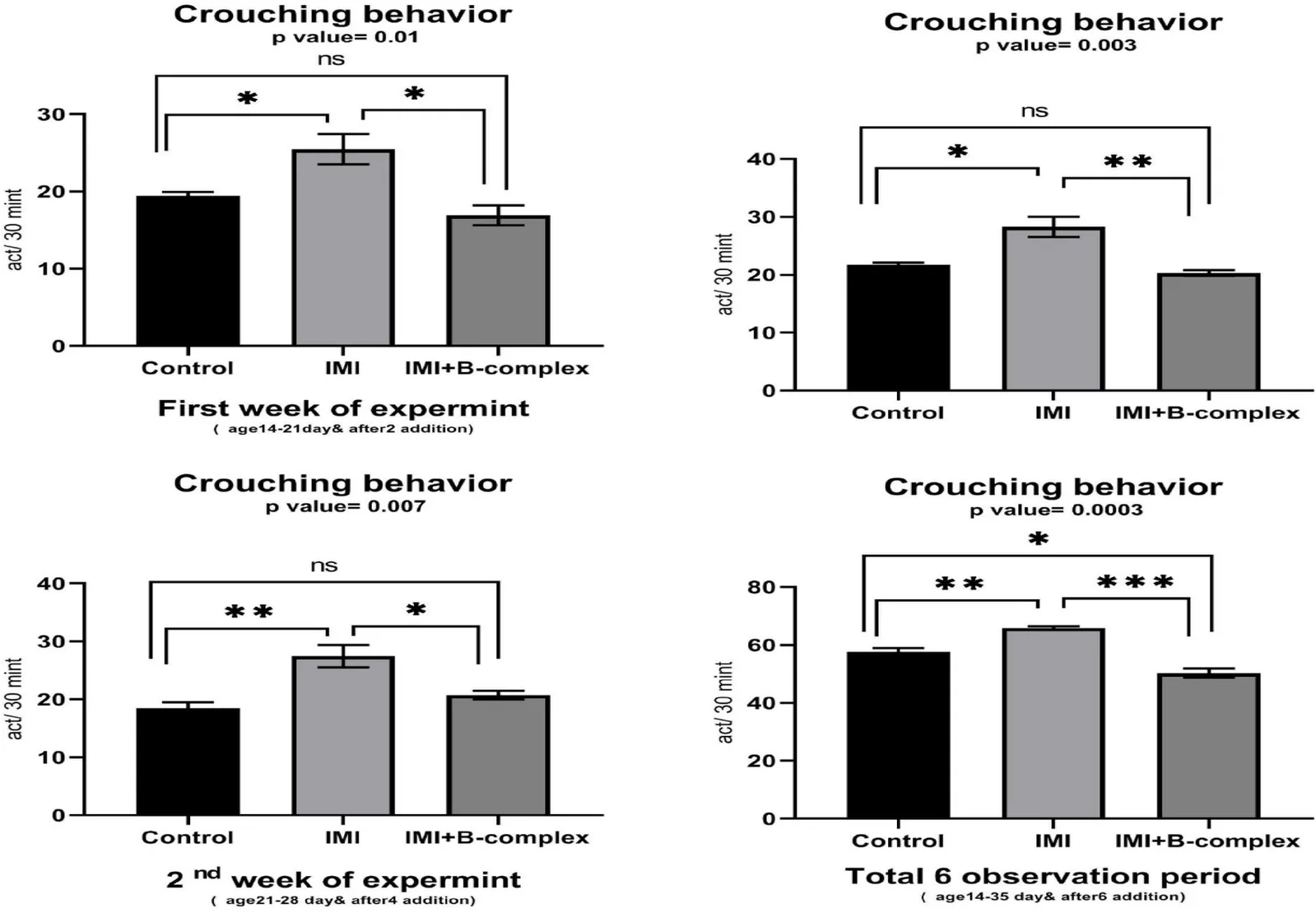
Effect of imidacloprid and vitamin b complex on quail crouching behavior.

Figures 1, 2, 3, 4 and 8 show significant reductions in ingestive (feeding and drinking), comfort, and locomotor (standing) behaviors. On the contrary, a significant increase in crouching behavior was observed, without showing any effect on walking behavior. Furthermore, tonic immobility test findings revealed a significant increase in fear duration and index in the quails orally exposed to IMI compared to the control birds.

### Effect of IMI exposure on Quail Performance

Oral administration of imidacloprid twice weekly at dose of 1/10 of LD50% dose of Imidacloprid (4.43 mg/kg from 70%) in water for one, 2, and 3 week for quails aged 14 days old (14 days till 35 day old) causing as indicative decrease in total feed intake, final body weight, body weight gain with remarkable increase in nourish conversion ratio as showed in Table 2.

**Table 2:** Effect of imidacloprid and b- complex effect on body performance.

	Control	Imidacloprid	Imidacloprid + B complex	|*p* value
*Body weight (gram)*				
Initial body weight (14 day old)	55.09 ± 1.28	55.5 ± 0.048	55.19 ± 1.2	0.870
Body weight (21 day old	113.49 ± 2.02	112.5 ± 1.5	113.67 ± 1.83	0.088
Body weight (28 day)	170.75 ± 5.12 a,b	165.6 ± 1.6 b	181.38 ± 1.9 a	0.037
Final body weight (35 day)	207.08 ± 4.2	192.67 ± 4.2	215.2 ± 2.7	0.0058
*Body weight gain (gram)*				
First body gain (21–14)	57.52 ± 0.79	57 ± 1.4	58.47 ± 0.6	0.615
Second body gain (28–21)	57.27 ± 4.05 b	53.11 ± 2.02 b	67.72 ± 2.27 a	0.031
Third body gain (35–28)	36.34 ± 1.05 a	27.05 ± 1.2 b	33.8 ± 1.8 b	0.005
Final body weight-intial body weight	151.13 ± 3.04 a	137.17 ± 1.84 b	160.43 ± 3.58 a	0.0027
*Feed intake (gram)*				
First experimental week (14–21 day)	128.7 ± 4.09	125.78 ± 8.5	117.5 ± 10.55	0.812
Second week (21–28)	122.37 ± 7.02	131.94 ± 8.6	153. ± 7.7	0.177
Third week (28–35)	145 ± 2.29 a	133.3 ± 2.02 b	143.5 ± 1.73 a	0.013
All feed intake (14–35 day)	396.29 ± 8.8	391.02 ± 5.8	413.5 ± 4.2	0.113
*Feed conversion ratio*				
First experimental week	2.24 ± 0.09	2.19 ± 0.26	2.01 ± 0.199	0.712
Second week	2.15 ± 0.13	2.48 ± 0.21	2.28 ± 0.19	0.485
Third week	4.0 ± 0.18b	4.9 ± 012a	4.23 ± 0.07 b	0.007
All feed intake/all body gain	2.62 ± 0.03 a	2.85 ± 0.01 b	2.58 ± 0.1a	0.002

### Effect of IMI exposure on Quail organs function and blood biochemistry

**Fig. 5:**
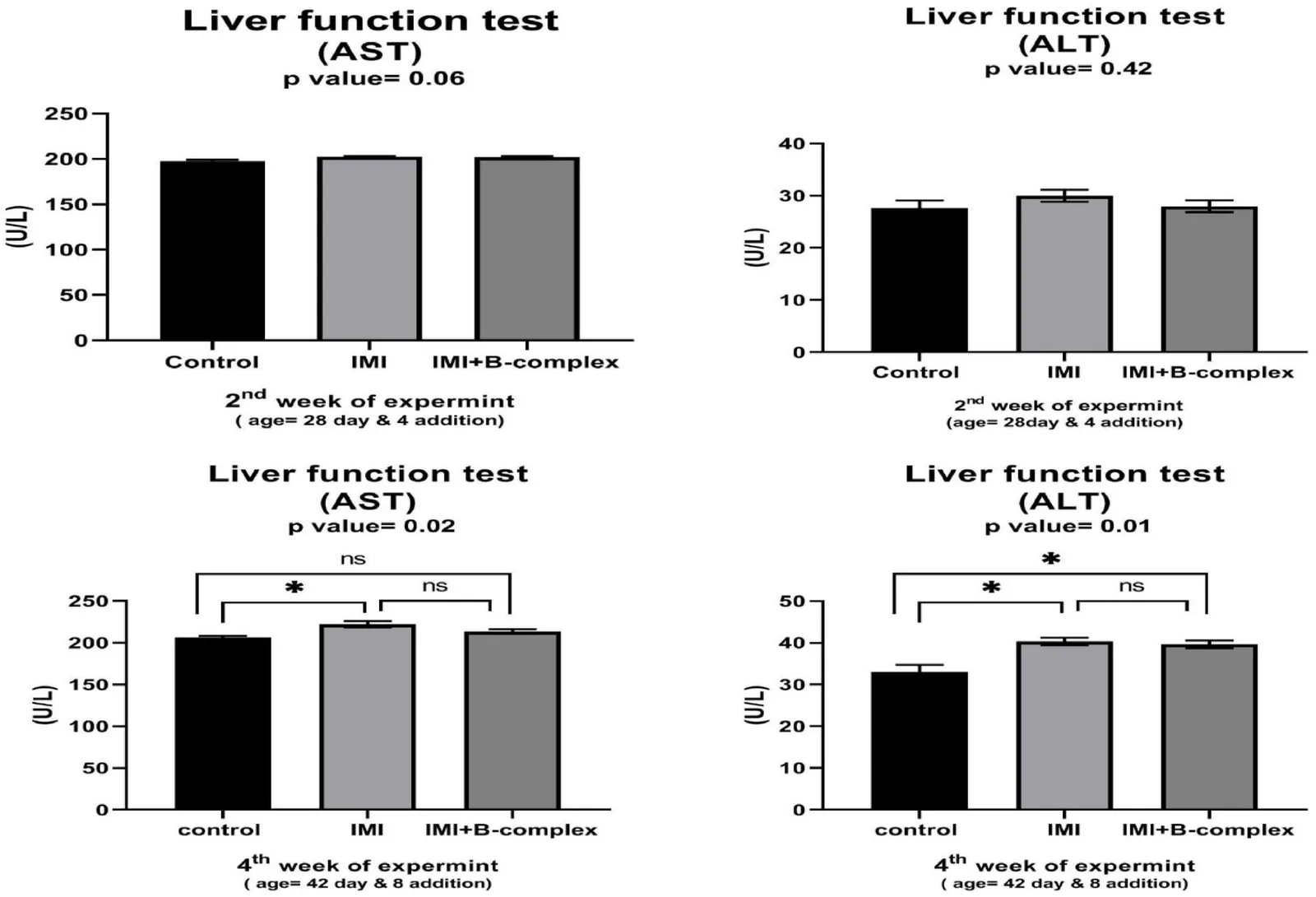
Effect of imidacloprid and vitamin b complex on quail liver function test.

**Fig. 6:**
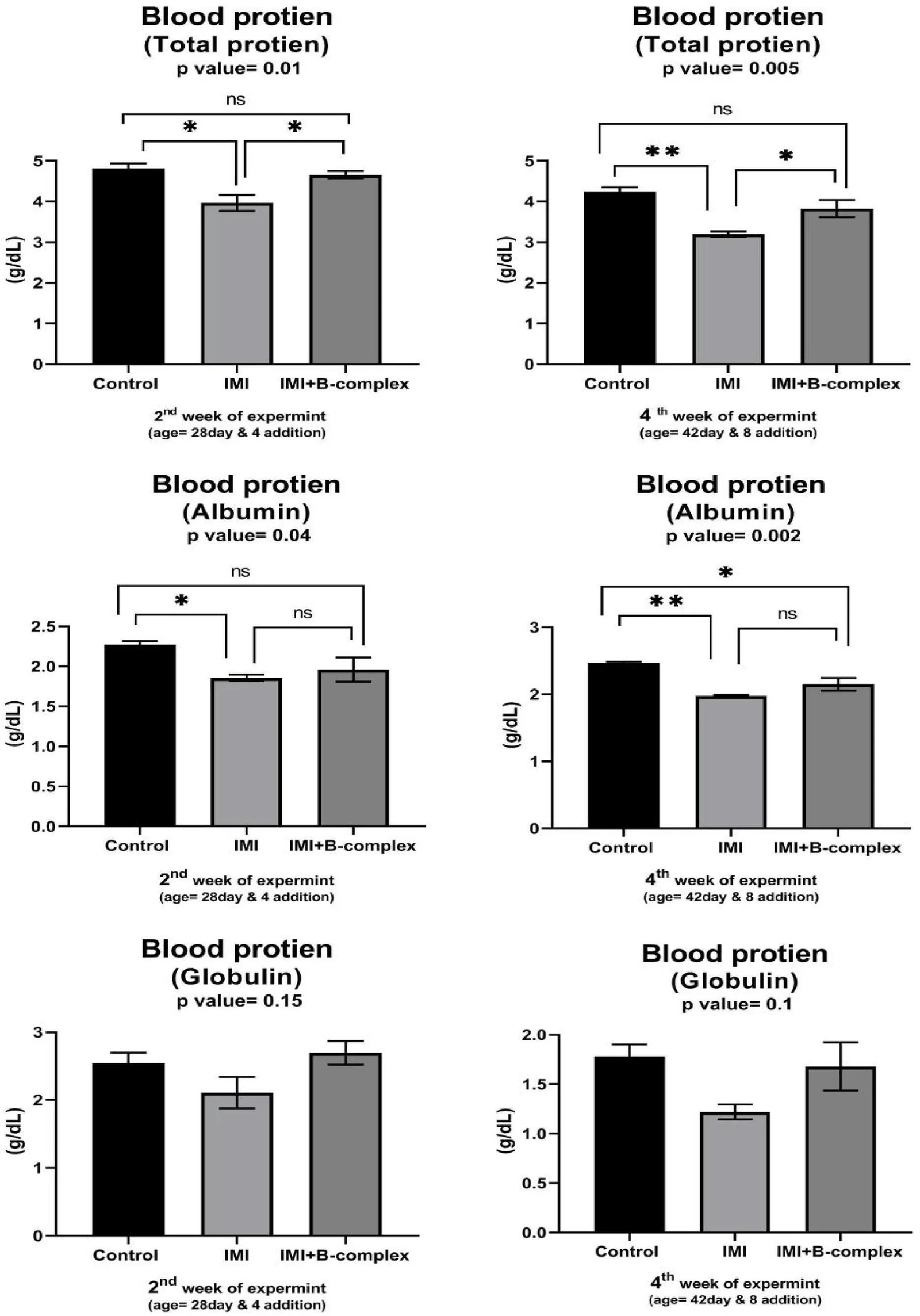
Effect of imidacloprid and vitamin b complex on quail major blood indicator.

**Fig. 7:**
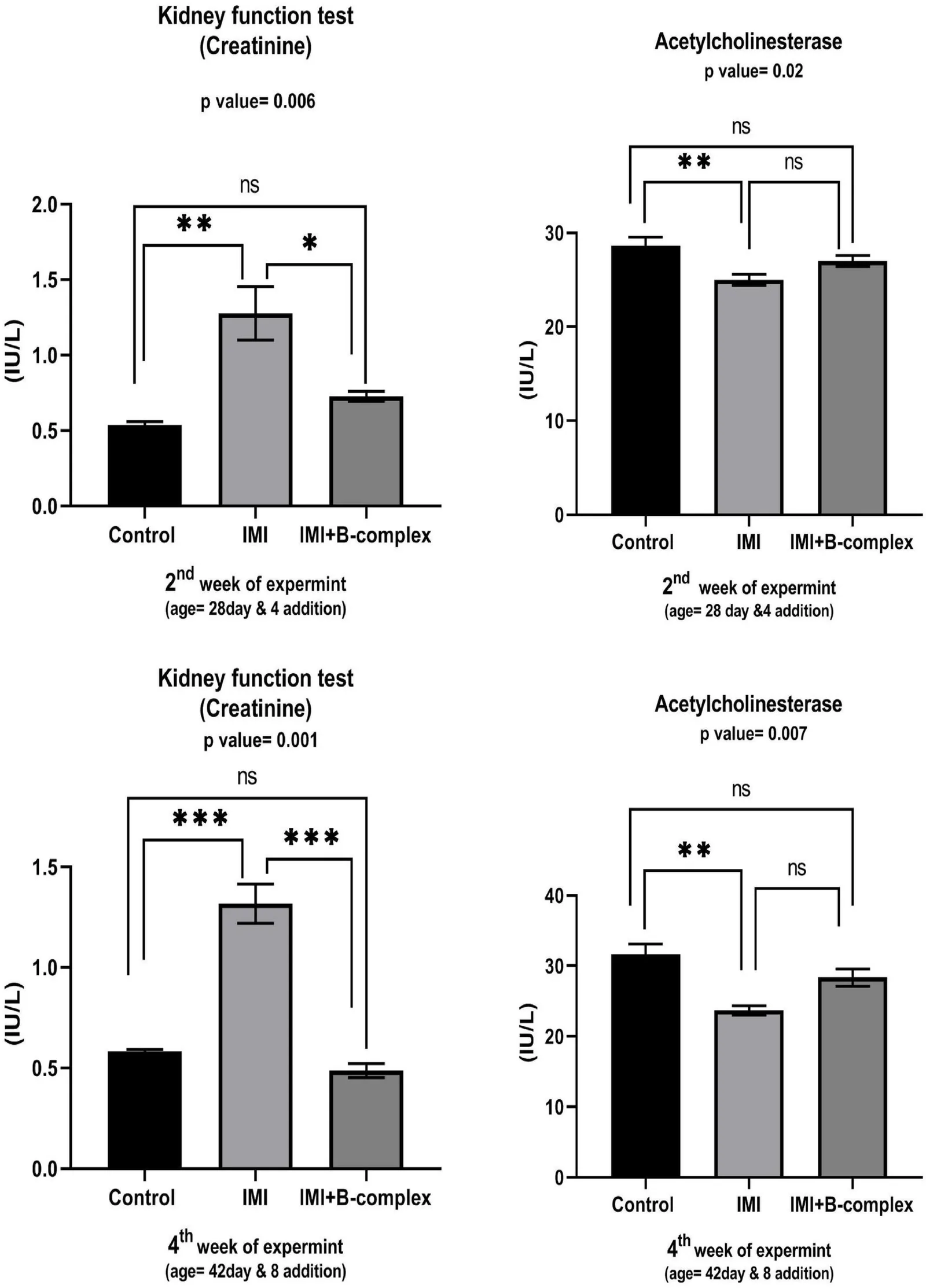
Effect of imidacloprid and vitamin b complex on quail neuromuscular indicator.

Blood sample biweekly or taken after 4 or 8 dose at the age 2 week of experiment (28 age old) and 4 week of experiment (42 day old age) showed that, a significant increase in liver function test (ALT and AST) and muscle atrophy indicator creatinine, while, there are a remarkable decrease in the entire protein, albumin, and ACH encounter some of these results can be solved by addition of B-complex as (creatinine and total protein) (Figs. 5, 6, 7).

### Effect of IMI exposure on the sciatic nerve

Transmission electron micrograph of sciatic nerve from control group showed normal morphological appearance of myelinated nerve, Schwann’s cells, and interstitial tissue (e). In the IM group, vacoulation and splitting of the myelin sheath, congestion and increase interstitium tissue were observed (a, & b) (Fig. 9). Marked deformity of nerve fibre was also seen in this group (c). Sciatic nerve from the IB group showed only moderate vacuolization of the myelin sheath (d) (Fig. 9).

**Fig. 8:**
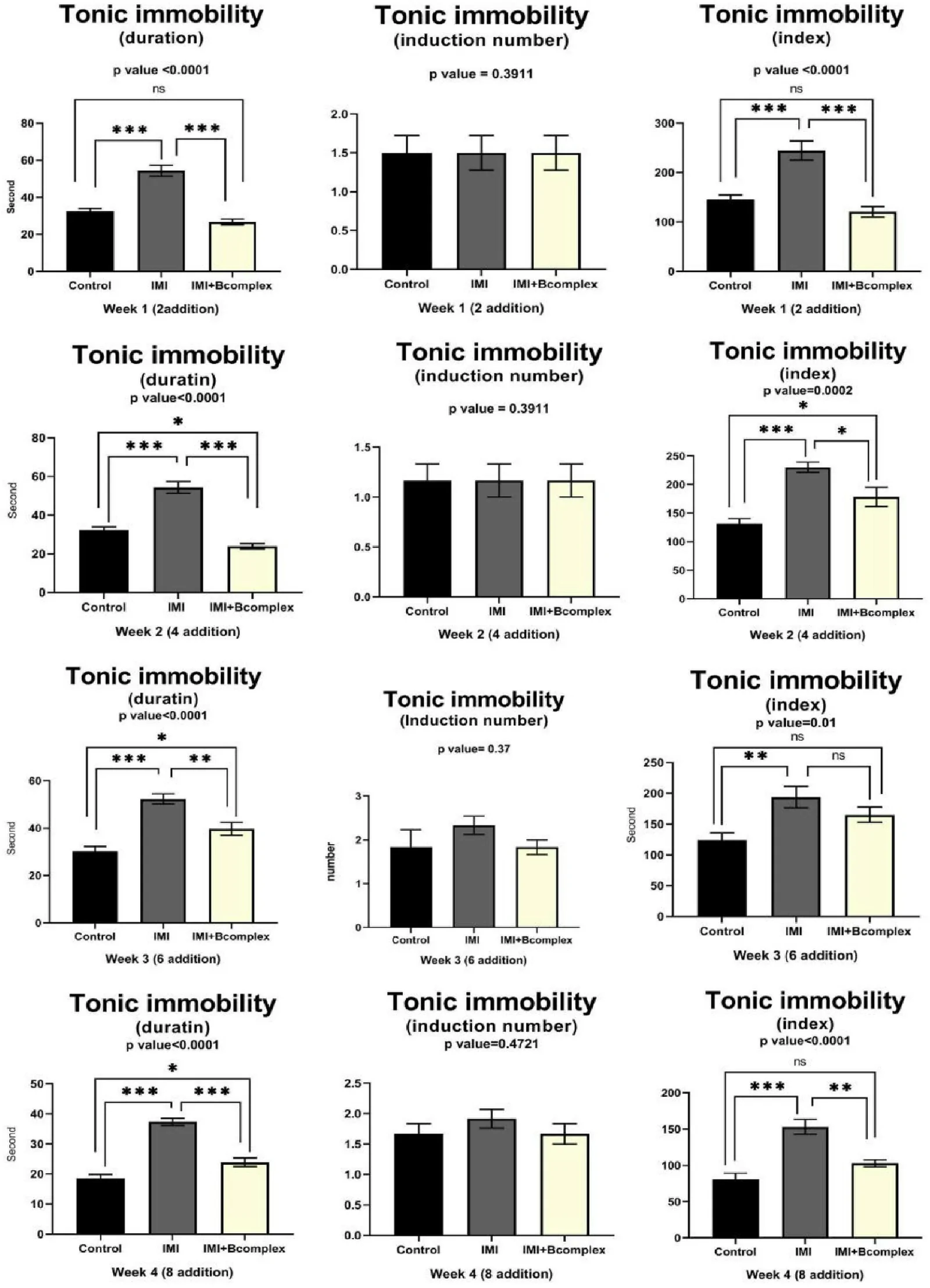
Effect of imidacloprid and vitamin b complex on quail welfare.

**Fig. 9:**
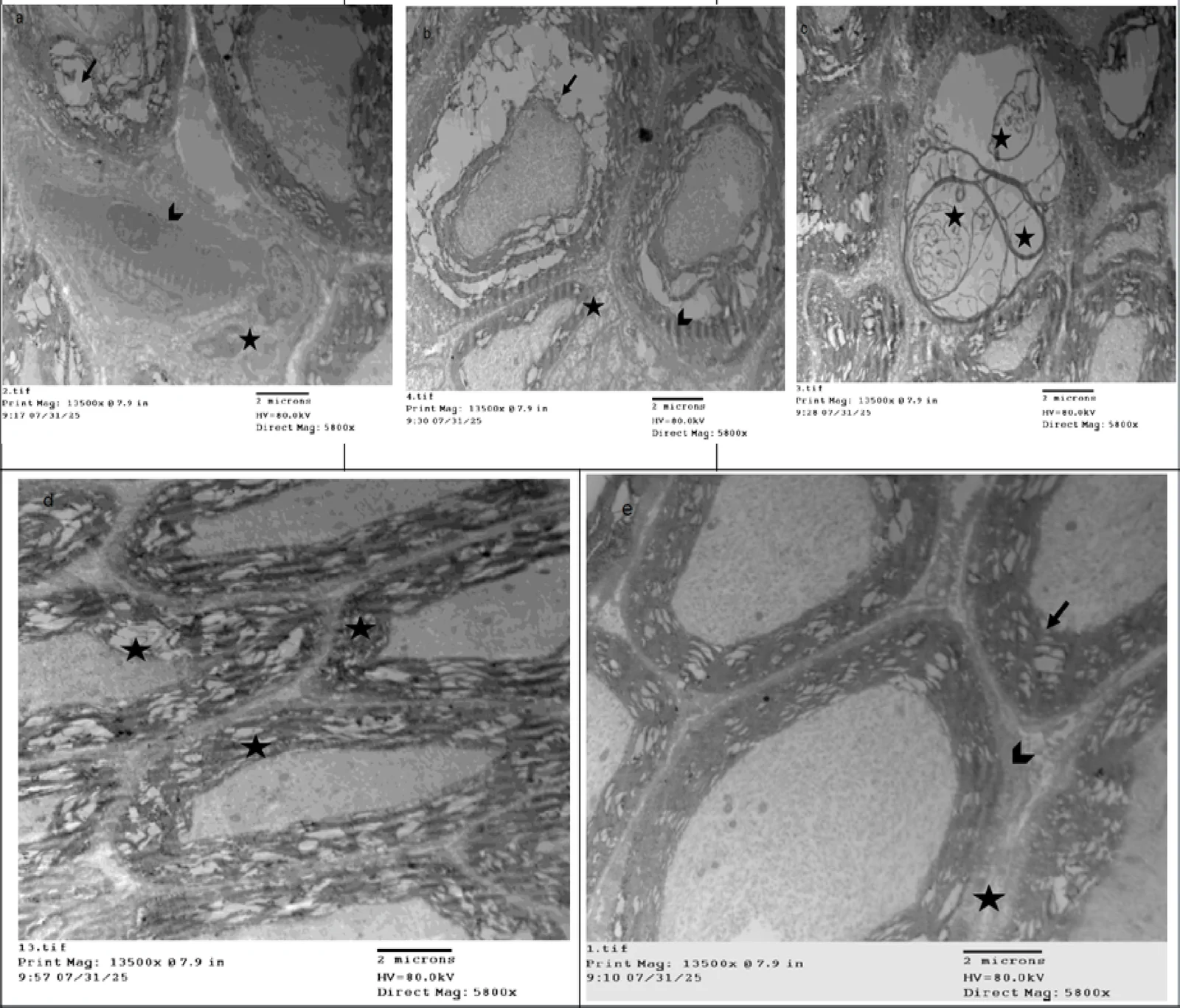
Effect of imidacloprid and vitamin b complex on quail sciatic nerve. (**a**) Transmission electron micrograph of sciatic nerve from Imidacloprid group showing vacoulation of myelin sheath (arrow), congestion (arrowhead) and increase interstitium tissue and sever deformity in the nerve fiber (star). (**b**) Transmission electron micrograph of sciatic nerve from IM group showing splitting of myelin sheath (arrow), sever myelin vacuolization (arrowhead) and increase interstitium tissue (star). (**c**) Transmission electron micrograph of sciatic nerve from IM group showing sever deformity in the nerve fiber (stars). (**d**) IMI + B-COMPLEX = Transmission electron micrograph of sciatic nerve of Imidacloprid and vit b -complex showing only moderate vacuolization 0f. myelin sheath (stars). (**e**) Transmission electron micrograph of sciatic nerve from control group showing normal morphological appearance of myelinated nerve (arrow), Schwann’s cells (arrowhead) and interstitium (star).

### Mitigation role of B-Complex

In contrast, the IB group showed moderate degeneration, with milder vacuolization and less pronounced myelin splitting. This suggests a partial protective effect of exposure to a lower- intensity toxic insult, resulting in a more preserved axonal structure.

## Discussion

The impact of neurotoxic pesticides, including IMI, on poultry can lead to diminished productivity and compromised health, which subsequently threatens the sustainability of the poultry industry^4^. Given the critical role of avian species in global meat production^30^, identifying interventions that can counteract the detrimental effects of such pesticides is essential^14^.

Consequently, this study was designed to evaluate the weekly and cumulative impacts of IMI on the behavior, welfare, performance, and selected organ functions of Japanese quail. Additionally, we investigated the potential ameliorative effects of a Vitamin B complex syrup (Becozem). The evaluation was conducted over a 4-week growth period, tracking the birds from 14 to 42 days of age, which represents a critical juvenile developmental stage for this species.

Behavior is widely considered a reliable proxy for overall health. It is highly sensitive compared to other life-history traits because it reflects the final, integrated outcome of various complex physiological and biochemical pathways. Consequently, behavioral changes serve as an effective tool for tracking environmental and toxicological perturbations^11^.

Moreover, feeding is a critical behavior directly tied to a bird’s need to meet its metabolic requirements^31^. It is a fundamental process for animal survival^32^ and is regulated by complex systems that control both central and peripheral sites, such as the brain, liver, and gastrointestinal tract^33^. The liver plays a central role in metabolism,it is responsible for the detoxification, excretion, synthesis, digestion, modulation, storage, and release of essential macronutrients like lipids, carbohydrates, and proteins^34^.

Exposure to imidacloprid (IMI) resulted in a significant reduction in ingestive behavior frequency (feeding and drinking), feed intake, body weight (BW), body weight gain (BWG), total protein, and albumin levels. Conversely, a significant increase was observed in the feed conversion ratio (FCR) as well as both aminotransferase (AST and ALT) levels.

These findings align with previous results reported by^12^ and Abd-Elhakim et al.^19^ for performance metrics,Eleiwa et al.^4^ and Osman et al.^35^ for behavioral alterations,Gul et al.^6,36^ for ingestive patterns, and Eleiwa et al.^4^, Zaahkook et al.^37^, Abotaleb et al.^38^, and Hassanen et al.^39^ for biochemical profiles, respectively.

Moreover, the non-significant IMI effect on feeding behavior data in first and second weeks of the experiment was consistent with the findings of Maria and Brodeur^6^.

The observed reduction in ingestive behavior frequency specifically feeding and drinking alongside the overall decrease in feed intake, can be attributed to a multi-faceted cascade of physiological, neurological, and metabolic disruptions induced by Imidacloprid (IMI) exposure. Centrally, neonicotinoids are well documented to disrupt core nutrient metabolism and compromise gut health in avian species^40^. This decline in consumption is initially driven by immediate post-ingestive distress and pronounced anorexic effects^5,6,12^. Mechanistically, IMI target postsynaptic nicotinic acetylcholine receptors (AChRs), inducing neurological alterations that impair the trigeminal sensorimotor system,this functionally impedes the birds’ mechanical capacity for seed grasping and beak manipulation^15,12^.

Concurrently, the deregulation of central appetite networks and the suppression of gastrointestinal motility prompt an acute loss of appetite^41,42^, which downstream severely compromises feed digestion and mucosal absorption within the avian gut^15^.

Beyond these behavioral and mechanical limitations, IMI interferes deeply with systemic metabolic pathways^14,43^, culminating in the impairment of cellular energy metabolism and nutrient assimilation^44^. This systemic pathology is closely linked to the toxicological burden exerted on the liver and kidneys^39^. Specifically, IMI-induced hepatocellular damage suppresses hepatic protein synthesis^45^, which aligns with the observed serum biochemical profile: a significant decline in total protein and albumin levels, coupled with a concomitant rise in aspartate aminotransferase (AST) and alanine aminotransferase (ALT) activities. Collectively, these findings demonstrate that IMI limits feed intake not merely through aversion, but through a compound effect of sensorimotor paralysis, gastrointestinal stasis, and severe metabolic hepatotoxicity.

Beyond direct ingestive inhibition, the observed degradation in avian performance metrics characterized by a decline in cumulative feed intake, suppressed final body weight, diminished body weight gain, and a elevated feed conversion ratio (FCR) can be attributed to the significant “metabolic cost” of xenobiotic detoxification. The physiological process of eliminating IMI requires substantial energy expenditure, shifting metabolic priority away from somatic growth and altering the homeostatic utilization of primary substrates including proteins, carbohydrates, and lipids^46^. Consequently, this systemic reallocation of energy toward detoxification pathways drives severe growth retardation and subsequent body weight deficits^6^. Furthermore, the elevation in (FCR) highlights a profound disruption in feeding efficiency. This compromised efficiency is partly mediated by behavioral changes, as IMI exposure extends the duration required for birds to handle and consume food^6^, effectively lowering the net energy gained per unit of foraging effort.

The weekly oscillations in the drinking behavior could be elucidated as a plan of the birds to pro tempore retrieve from IMI consequences by decreasing their water or meal consumption and thence the IMI intake^6^, birds lessen their water drink due to the anorexic consequences of IMI. That being so, the quantity of IMI passing into the creature would be lowered or pro tempore stopped, easing the excretion of the diffusing IMI that lasts from 12 to 48 h^12,47^.

On the other side, the water amount was non-significant, with an increased rate or frequency of drinking behavior affected, possibly due to birds beginning to organise the intake of IMI by lowering the quantity of water consumed by stake whilst increasing the number of drinking stakes throughout the day. The birds consuming the same quantity of water for a longer time would permit the creature to partly excrete the consumed IMI, with the same line of the previous findings in the case of seed consumption containing IMI^6^. This phenomenon is sufficient to decrease the poisoning marks that are linked to exposure for a short time^5,15,12^.

Locomotion behavior represents a dynamic equilibrium between resting durations and active displacement, serving to satisfy essential behavioral and physiological prerequisites^48^. As a fundamental output of central nervous system (CNS) function, locomotive responses to environmental stimuli are critical for animal survival^49^. These motor patterns facilitate vital adaptive functions, such as foraging, mate localized searching, and predator avoidance^50^. Furthermore, quantified baseline locomotion provides critical insights into broader neurobehavioral states, directly reflecting home-cage activity levels, fear responses, social interactions, and systemic stress reactivities^51^.

The present study demonstrated a significant reduction in active locomotor behavior (specifically standing durations) alongside a concomitant suppression of acetylcholinesterase (AChE) levels in IMI-exposed quails relative to the control group. Conversely, exposure to oral IMI induced a significant elevation in both crouching behavior and serum creatinine levels, whereas walking behavior remained statistically unaffected. This distinct behavioral and biochemical profile strongly aligns with earlier reports. Specifically, the observed alterations in general locomotion and standing time corroborate the findings of Maria and Brodeur^6^ and Abd-Elhakim et al.^19^. The inhibition of AChE activity matches the neurotoxic profiles described by Ali et al.^52^ and Kamdem et al.^53^. Furthermore, the compensatory increase in crouching behavior is consistent with the sedentary postures reported by^6^ and Abd-Elhakim et al.^19^, while the elevation in creatinine levels confirms the nephrotoxic impacts previously identified by Hassanen et al.^39^ and Abd-Elhakim et al.^19^.

Locomotion and motor behavior emerge from integrated, reciprocal interactions between the nervous, musculoskeletal, circulatory, and respiratory systems and the external environment, generating complex temporal dynamics^54^. Consequently, the locomotor dysfunction and marked reduction in active behaviors observed in the present study can be characterized through a tripartite physiological framework consisting of musculoskeletal degradation, structural peripheral neurotoxicity, and biochemical disruption of cholinergic signaling.

Addressing the musculoskeletal component, the notable elevation in serum creatinine a primary byproduct of skeletal muscle creatine metabolism and a critical biomarker of nephrotoxicity^55^ strongly points to underlying, severe myodegeneration. This skeletal muscle damage is likely exacerbated by ongoing physical exertion under conditions of severe cellular dehydration^56^. Within the context of the present study, this state of systemic dehydration is a direct pathological secondary outcome of the down-regulated drinking behavior and restricted fluid intake documented earlier in the IMI-exposed cohorts, establishing a clear link between ingestive aversion and physical motor decline.

Compounding this muscular degradation is structural damage occurring at the nervous level. The histopathological evidence of peripheral nerve lesions obtained in this study strongly corroborates the established neurotoxic profile of IMI. This structural degeneration closely matches the findings of Wankhede et al.^57^, who reported apparent sciatic nerve bundle degeneration in Japanese quails following dietary exposure to 50 ppm IMI for 28 days. Central neuronal degeneration and encephalic necrosis induced by IMI^17^ can extend peripherally to major nerve pathways, such as the sciatic nerve. Downstream, this severe axonal pathology impairs sensorimotor coordination, suppresses basic exploratory drive, and diminishes key electrophysiological parameters specifically reducing the action potential velocity, amplitude, and latency within the sciatic nerve bundle^58^.

At the synaptic level, these mechanical and structural deficits are driven by acute biochemical lesions within cholinergic pathways. Chronic IMI exposure triggers an absolute suppression of acetylcholinesterase (AChE) activity, driven by a dual-action neurotoxic mechanism involving oxidative stress, mitochondrial failure, and localized neuroinflammation^52,53^. Mechanistically, IMI directly binds to the active catalytic site of AChE, as demonstrated by computational modeling, which structurally interferes with normal enzyme kinetics^53^. Concurrently, severe oxidative damage compromises neural membrane integrity and disrupts homeostatic cholinergic signaling pathways^59^. The resulting repression of AChE prevents the rapid enzymatic degradation of acetylcholine (ACh), prompting its pathognomonic accumulation within the synaptic cleft. This leads to chronic overstimulation of postsynaptic nicotinic acetylcholine receptors (nAChRs), which ultimately exhausts synaptic transmission across both central networks and peripheral neuromuscular junctions, culminating in profound neuromuscular fatigue and motor failure^60^.

Conversely, the observed locomotor dysfunction—characterized by a significant increase in crouching postures alongside a parallel decrease in standing and walking behaviors reflects an adaptive and pathological behavioral modification. This behavioral shift can be attributed to immediate post-digestive distress, which prompts the birds to remain sedentary in distinct cage areas with heavily restricted activity rates^15^. Beyond localized distress, these postural alterations are driven by systemic neurological adjustments induced by IMI and its metabolites. Upon binding to postsynaptic nicotinic receptors, these compounds impair normal neurotransmission, giving rise to clinical manifestations such as reduced mobility and persistent tremors^15^,^12^.

Mechanistically, this process disrupts the homeostatic synchronization between neuronal signaling and skeletal muscle responses, a consequence of the marked alteration in acetylcholinesterase (AChE) activity that ultimately modifies general locomotor attitudes and voluntary activity^61^. Because IMI structurally mimics endogenous acetylcholine (ACh), it binds tenaciously to nicotinic acetylcholine receptors (nAChRs), inducing sustained, unregulated receptor activation. This persistent stimulation completely disrupts normal cholinergic neurotransmission, triggering an excitotoxic pathway that ultimately culminates in advanced neuronal dysfunction and structural degeneration^35^.

Feather preening and related grooming actions serve as primary behavioral indicators of a positive comfort state in avian species^62^. Conversely, diminished frequencies of these comfort activities are inversely correlated with elevated baseline fear levels^63^.

The data obtained in the present study revealed a significant reduction in comfort behaviors specifically grooming and feather ruffling concurrent with a significant escalation in fear-related metrics, including tonic immobility duration, index, and induction number. This behavioral profile is in complete alignment with previous literature, where the reduction in comfort behavior and the concomitant amplification of fear responses corroborate the findings of Janner et al.^64^, Abd-Elhakim et al.^19^, respectively.

Physiologically, this marked behavioral shift may be attributed to xenobiotic-induced neurobehavioral toxicity. Exposure to imidacloprid has been documented to trigger abnormal repetitive movements that supplant normal maintenance behaviors^64^, directly resulting in altered or fragmented grooming patterns^65^. Furthermore, the exacerbation of fear-related behaviors manifested through the prolonged tonic immobility response underscores a state of heightened systemic stress and central nervous system vulnerability induced by neonicotinoid neurotoxicity, which severely compromises the birds’ psychological and physiological welfare.

The marked escalation in tonic immobility duration and index indicative of heightened baseline fearfulness and anxiety states can be traced to a complex interplay of transcriptional deregulation and structural encephalic injury. At the molecular level, this neurobehavioral shift is closely linked to the up-regulation of key apoptosis-related genes, specifically Caspase-3 and P21, occurring alongside a sweeping transcriptional down-regulation of genes critical to homeostatic neurological architecture^19^.

Among these, the suppression of brain-derived neurotrophic factor (BDNF) mRNA expression stands as a pivotal driver of the observed pathologies. Because BDNF is structurally indispensable for neuronal survival, neurogenesis, synaptic plasticity, and precise neurotransmitter modulation^66^, its exhaustion directly correlates with anxiety-related behavioral phenotypes, elevated stress susceptibility, and impaired fear extinction pathways^67^. This aligns with broader toxicological paradigms established by Rodriguez-Carrillo et al.^68^, who reported a distinct negative association between chronic pesticide exposure, diminished BDNF expression, and subsequent neurobehavioral disorders.

This genetic vulnerability is further compounded by the simultaneous down-regulation of glucagon-like peptide-1 (GLP-1), peroxisome proliferator-activated receptor alpha (PPARA), and peroxisome proliferator-activated receptor gamma coactivator 1-alpha (PGC-1α). Within the central nervous system, GLP-1 exerts vital neuroprotective and neurogenic properties, mitigates programmed cell death, and modulates cognitive and behavioral adaptations^69^. Concurrently, the suppression of PPARA compromises cellular antioxidative and anti-inflammatory shielding mechanisms while actively disrupting fatty acid transport, lipid homeostatic pathways, and mitochondrial bioenergetics^70^.

Because PGC-1α plays a foundational role in driving cellular differentiation and mitochondrial biogenesis, its down-regulation strongly correlates with heightened clinical anxiety, motor deficits, and neuroinflammatory cascades^71,72^. Taken together, the synchronized transcriptional repression of BDNF, GLP-1, PPARA, PGC-1α, and GAPDH in the brains of IMI-exposed quails provides a robust molecular signature of severe cognitive and functional brain impairment.

Ultimately, these downstream transcriptional aberrations map directly onto macroscopic and structural encephalopathic changes. Due to its lipophilic properties, IMI readily traverses the blood–brain barrier^73^. Upon entering the central nervous system, it stimulates cytochrome P450 (CYP450) enzymatic pathways, generating an acute influx of reactive oxygen species (ROS)^74^. This localized oxidative stress rapidly depletes endogenous antioxidant enzyme defenses while simultaneously driving extensive lipid peroxidation of neuronal membranes^75^. The resulting oxidative damage leads to visible histopathological and encephalopathic degeneration across multiple brain regions, supplying the definitive structural basis for the profound behavioral anomalies and hyper-fearful states observed in the exposed birds^19^.

The improved performance of the IB group may reflect an early protective or stimulatory effect of the vitamin B complex. B-complex vitamins, mainly B1 (thiamine), B6 (pyridoxine), and B12 (cobalamin), are momentous cofactors in carbohydrate metabolism, amino acid metabolism, and neurological function. The B vitamins perform essential tasks in the metabolism^76^. These vitamins may enhance neural signalling and digestive enzyme activity, potentially improving appetite and nutrient assimilation under mild toxic stress^77^. B-complex vitamins, can mitigate the adverse effects of pesticides in birds by enhancing antioxidant defence mechanisms and promoting metabolic recovery^4^, supporting metabolic enzyme function. Chronic oxidative and neurological stress from imidacloprid likely overwhelmed the physiological buffering capacity of the vitamins^14^.

In summary, Vitamin B complex temporarily supports quail’s performance under pesticide stress; it does not fully counteract the long-term toxic effects of imidacloprid on neural and gastrointestinal systems. Just as B vitamins are hydrosoluble that denotes the body doesn’t store them, so they require replaced quotidian^78^. Our findings emphasise the need for longer-term antioxidant or detoxification strategies to protect avian species from environmental contaminants.

Interestingly, co-treatment with Vitamin B complex (IB) numerically but non-significantly mitigated these hepatic enzyme elevations as AST and ALT activities sustain that vitamin B6. Riboflavin, folic acid and choline affect the storage of vitamin B12 in the liver, which may be helpful in mitigating hepatic inflammation and diseases^79^.

The protective role of B vitamins, especially B6 (pyridoxine) and B12, is well documented. These micronutrients support cellular resilience through a variety of mechanisms: Neutralization of ROS and reduction of lipid peroxidation, preserving membrane integrity enhancement of mitochondrial enzymatic activity, aiding in energy homeostasis and facilitating cell survival under toxic stress^80^.

While the protein levels were consistently, lower than those in the IM-only group in the later stages. This could suggest that although the vitamin B complex may help dampen inflammatory responses, it does not fully counteract the suppressive effect of long-term imidacloprid exposure on protein biosynthesis. Similar notices were published by Bashandy et al.^81^, who noted partial biochemical restoration with B-vitamin co-treatment yet persistent signs of hepatic compromise.

The protective mechanism of the vitamin B complex can be attributed to several physiological functions. Vitamins B6 (pyridoxine) and B12, in particular, are known to support mitochondrial integrity and overall renal cellular health. They enhance oxidative defence systems, reduce inflammation, and improve cellular energy metabolism. Vitamin B6 has shown protective effects in renal injury models, such as diabetic nephropathy and chemically induced toxicity, through its role in mitigating oxidative stress, reducing pro-inflammatory cytokines, and preserving renal perfusion^82^. Together, these findings underscore the value of vitamin B complex as a protective adjunct in mitigating early renal functional alterations associated with pesticide exposure.

Likely due to cholinergic neurotoxicity induced by imidacloprid. In the system of the central nervous, this insecticide acts as an nAChRs agonist, leading to overstimulation, energy depletion, and oxidative stress^43^. In contrast, the IB group-maintained AChE levels near baseline throughout the experiment. This consistent enzymatic preservation implies a protective effect of the vitamin B complex^83^. This is supported by its known biological functions, such as vitamins B6 and B12 being essential for myelin synthesis, neurotransmitter modulation, and cellular repair processes. Besides that, the B complex group collectively helps reduce oxidative damage and neuro-inflammatory responses, both of which are implicated in AChE inhibition. Additionally, B vitamins support choline metabolism and methylation cycles, both critical for normal neural function and AChE stability^84^.

B vitamins play fundamental tasks in energy metabolism, and nervous system maintenance, which might counteract the neurotoxic stress induced by imidacloprid exposure^76^. Obtained data illustrated the treatment with IM in combination with vitamin B showed amelioration of acetylcholine activity at 4 weeks of exposure and returned to normal values.

## Conclusion

In conclusion, long-term exposure to sub-lethal doses of imidacloprid in poultry production causes a comprehensive deterioration across behavioral, biochemical, and histopathological indices, ultimately undermining overall physiological performance and avian welfare. Consequently, incorporating a vitamin B complex into poultry management protocols is strongly recommended as a practical and accessible dietary intervention to actively reduce the unavoidable environmental footprint and adverse impacts of neonicotinoid exposure in livestock operations.

## Supplementary Information

Below is the link to the electronic supplementary material.

Supplementary Material 1

Supplementary Material 2

## Data Availability

Requests for materials should be addressed to corresponding author.

## References

[1] Peterson, E. K. et al. Integrative behavioral ecotoxicology: Bringing together fields to establish new insight to behavioral ecology, toxicology, and conservation. *Curr. Zool.* **63**(2), 185–194 (2017). 29491976 10.1093/cz/zox010 PMC5804166

[2] Yan, X.-T., Cai, Y.-Y., Zhang, Q.-Q., Guo, Z. & Ying, G.-G. Neonicotinoid insecticides in a large-scale agricultural basin system use, emission, transportation, and their contributions to the ecological risks in the Pearl River Basin, China. *Sci. Total Environ.* **948**, 174392 (2024). 38955277 10.1016/j.scitotenv.2024.174392

[3] Grzinic, G. et al. Intensive poultry farming: A review of the impact on the environment and human health. *Sci. Total Environ.* **858**, 160014 (2023). 36368402 10.1016/j.scitotenv.2022.160014

[4] Eleiwa, N. Z. et al. Dietary curcumin modulating effect on performance, antioxidant status, and immune-related response of broiler chickens exposed to imidacloprid insecticide. *Animals* **13**(23), 3650 (2023). 38067001 10.3390/ani13233650 PMC10705146

[5] Addy-Orduna, L. M., Brodeur, J. C. & Mateo, R. Oral acute toxicity of imidacloprid, thiamethoxam and clothianidin in eared doves: A contribution for the risk assessment of neonicotinoids in birds. *Sci. Total Environ.* **650**, 1216–1223 (2019). 30308809 10.1016/j.scitotenv.2018.09.112

[6] Poliserpi, M. & Brodeur, J. C. Behavioral and physiological changes in the passerine *Agelaioides badius* following the ingestion of coated seeds with imidacloprid in a 30-day experiment. *Sci. Total Environ.* 10.1016/j.scitotenv.2023.167078 (2023). 37717765

[7] Gibbons, D., Morrissey, C. & Mineau, P. A review of the direct and indirect effects of neonicotinoids and fipronil on vertebrate wildlife. *Environ. Sci. Pollut. Res. Int.* **22**(1), 103–118 (2015). 24938819 10.1007/s11356-014-3180-5 PMC4284370

[8] Karahan, A. et al. Sublethal imidacloprid effects on honeybee flower choices when foraging. *Ecotoxicol.* **24**(9), 2017–2025 (2015). 10.1007/s10646-015-1537-2 26415950

[9] Wang, X. et al. Mechanism of neonicotinoid toxicity: Impact on oxidative stress and metabolism. *Annu. Rev. Pharmacol. Toxicol.* **58**, 471–507 (2018). 28968193 10.1146/annurev-pharmtox-010617-052429

[10] Ford, A. T. et al. The role of behavioral ecotoxicology in environmental protection. *Environ. Sci. Technol.* **55**, 5620–5628. 10.1021/acs.est.0c06493 (2021). 33851533 PMC8935421

[11] Moreau, J., Monceau, K., Gonnet, G., Pfister, M. & Bretagnolle, V. Organic farming positively affects the vitality of passerine birds in agricultural landscapes. *Agric. Ecosyst. Environ.* **336**, 108034. 10.1016/J.AGEE.2022.108034 (2022).

[12] Poliserpi, M. B., Cristos, D., Pérez-Iglesias, J. M. & Brodeur, J. C. Tissue distribution and sublethal effects of imidacloprid in the South American grayish baywing (*Agelaioides badius*). *Chemosphere* **284**, 131327 (2021). 34216921 10.1016/j.chemosphere.2021.131327

[13] Moreau, J. et al. Pesticide impacts on avian species with special reference to farmland birds: A review. *Environ. Monit. Assess.* 10.1007/s10661-022-10394-0 (2022). 36107257

[14] Eid, Y. Z. et al. Mitigation of imidacloprid toxicity in poultry chicken by selenium nanoparticles: Growth performance, lipid peroxidation, and blood traits. *Biol. Trace Elem. Res.* **201**(11), 5379–5388 (2023). 36790585 10.1007/s12011-023-03592-5 PMC10509070

[15] Franzen-Klein, D. et al. Evaluation of neurobehavioral abnormalities and immunotoxicity in response to oral imidacloprid exposure in domestic chickens (*Gallus gallus domesticus*). *J. Toxicol. Environ. Health A* **83**(2), 45–65 (2020). 32024444 10.1080/15287394.2020.1723154 PMC8087242

[16] Abd-Elhakim, Y. M. et al. Thymol abates the detrimental impacts of imidacloprid on rat brains by lessening oxidative damage and apoptotic and inflammatory reactions. *Chem.-Biol. Interact.* **383**, 110690 (2023). 37648049 10.1016/j.cbi.2023.110690

[17] Abd-Elhakim, Y. M., Mohammed, H. H. & Mohamed, W. A. M. Imidacloprid impacts on neurobehavioral performance, oxidative stress, and apoptotic events in the brain of adolescent and adult rats. *J. Agric. Food Chem.* **66**(51), 13513–13524 (2018). 30501185 10.1021/acs.jafc.8b05793

[18] Hassanen, E. I. et al. Potential mechanisms of imidacloprid-induced neurotoxicity in adult rats with attempts on protection using *Origanum majorana* L. oil/extract: In vivo and in silico studies. *ACS Omega* **8**(21), 18491–18508 (2023). 37273614 10.1021/acsomega.2c08295 PMC10233680

[19] Abd-Elhakim, Y. M. et al. Moringa oleifera leaves powder mitigates imidacloprid-induced neurobehavioral disorders and neurotoxic reactions in broiler chickens by regulating the caspase-3/Hsp70/PGC-1α Pathway. *J. Agricult. Food Chem.* **73**(13), 8040–8053 (2025). 10.1021/acs.jafc.5c00252 40110847

[20] de Faria, D. B. G. et al. Behavioral changes in Japanese quails exposed to predicted environmentally relevant abamectin concentrations. *Sci. Total Environ.* **636**, 1553–1564 (2018). 29913616 10.1016/j.scitotenv.2018.04.293

[21] Abdelfattah, E. M., Ahmed, E. A., Fathi, A. E., Khalil, F. A. & Elgazzar, M. M. Environmental management and rearing facilities impact on growth performance of quail chicks. *Poult Sci J.* **12**(1), 45–52 (2023).

[22] Lehner, P. N. Sampling methods in behavior research. *Poult. Sci.* **71**(4), 643–649 (1992). 1594516 10.3382/ps.0710643

[23] Campbell, D. L., Dickson, E. J. & Lee, C. Application of open field, tonic immobility, and attention bias tests to hens with different ranging patterns. *PeerJ* **7**, e8122 (2019). 31788364 10.7717/peerj.8122 PMC6882422

[24] Mahmoud, U. T., Tuyttens, F. A., Farghal, M., Abd El-Reda, G. & Shoukary, R. D. E. Impact of restraining position on tonic immobility in broiler chickens and mule ducks. *Appl. Anim. Behav. Sci.* **282**, 106499 (2025).

[25] Kraimi, N. et al. Absence of gut microbiota reduces emotional reactivity in Japanese quails (*Coturnix japonica*). *Front. Physiol.* **9**, 603. 10.3389/fphys.2018.00603 (2018). 29881357 PMC5976779

[26] Shahdan, I. A., Regenstein, J. M. & Rahman, M. T. Critical limits for the control points for halal poultry slaughter. *Poult. Sci.* **96**(6), 1970–1981 (2017). 27965405 10.3382/ps/pew427

[27] Riaz, M., Asghar, A., Ali, F., Ahmad, S. & Shah, M. A. Evaluation of different slaughter methods on welfare and meat quality in poultry. *Vet. World.* **14**(7), 1745–1753 (2021).

[28] Boduç, E. & Allahverdi, E. Macroanatomical investigation of sciatic nerve in rat and quail as a model for experimental medical studies: A comparative study of anatomy: Macroanatomical investigation of sciatic nerve. *Med. Sci. Discov.* **10**, 765–771. 10.36472/msd.v10i10.1052 (2023).

[29] Bozzola, J. J. & Russell, L. D. *Electron microscopy: Principles and techniques for biologists* (Jones & Bartlett Learning, 1999).

[30] Abd El-Maguid, D. S. et al. Combined exposure to monosodium glutamate and sodium sulfite in broiler diets: Impacts on growth, oxidative stress, and tissue integrity. *Res. Vet. Sci.* **204**, 106131 (2026). 41812339 10.1016/j.rvsc.2026.106131

[31] Lovette, I. J. & Fitzpatrick, J. W. *Cornell lab of ornithology’s handbook of bird biology* (Wiley, 2016).

[32] Tachibana, T. & Tsutsui, K. Neuropeptide control of feeding behavior in birds and its difference with mammals. *Front. Neurosci.* **10**, 485. 10.3389/fnins.2016.00485 (2016). 27853416 PMC5089991

[33] Pu, S. et al. The relation between liver damage and reproduction in female Japanese quail (*Coturnix japonica*) exposed to high ambient temperature. *Poult. Sci.* **99**(9), 4586–4597. 10.1016/j.psj.2020.05.025 (2020). 32868003 PMC7598027

[34] Zaefarian, F., Abdollahi, M. R., Cowieson, A. & Ravindran, V. Avian liver: The forgotten organ. *Animals (Basel).* **9**(2), 63. 10.3390/ani9020063.PMID:30781411;PMCID:PMC6406855 (2019). 30781411 PMC6406855

[35] Osman, K. A., Shaaban, M. M. & Ahmed, N. S. Biomarkers of imidacloprid toxicity in Japanese quail, *Coturnix coturnix japonica*. *Environ. Sci. Pollut. Res. Int.* **30**(3), 5662–5676 (2023). 35980528 10.1007/s11356-022-22580-1

[36] Gul, S. T. et al. Amelioration of toxicopathological effects of thiamethoxam in broiler birds with vitamin E and selenium. *Toxin Rev.* **41**(1), 218–228 (2022).

[37] Zaahkook, A. F., El-Masry, H., Delgado, M. & Nair, V. Pesticide-induced biochemical changes in poultry. *Vet. Toxicol. J.* **17**(4), 267–275 (2009).

[38] Abotaleb, A., AboSalem, M., Elshewy, E. A. & Abdeen, A. Alleviation of imidacloprid-induced oxidative stress and immune damage by *Spirulina platensis* in broiler chickens. *Benha Vet. Med. J.* **40**, 144–148 (2021).

[39] Hassanen, E. I., Abd El-Aziz, R. M., El-Gohary, M. I. & Soliman, M. M. Protective effects of antioxidants against imidacloprid-induced hepatotoxicity in rats. *Environ. Toxicol.* **37**(3), 481–491 (2022).

[40] Paleolog, J., Wilde, J., Gancarz, M. & Strachecka, A. Imidacloprid decreases the total energy production in western honeybees even though, in sublethal doses, it increased the values of six of the nine compounds in the respiratory and citric cycle. *bioRxiv [Preprint].* 10.1101/2025.03.21.123456 (2025). PMC12212523 40591539

[41] Li, X., Zhang, Y., Chen, L., Wang, J. & Liu, H. Pesticide effects on food consumption and body weight. *J. Agric. Toxicol.* **29**(1), 15–21 (2007).

[42] Ravikanth, V., Lakshman, M., Madhuri, D. & Kalakumar, B. Haematological alterations in broilers administered with imidacloprid and spinosad and its amelioration with vitamin E and silymarin. *Int. J. Curr. Microbiol. Appl. Sci.* **6**(4), 496–500 (2017).

[43] Ibrahim, M. I. A., Phaswane, R. M., Lensink, A. V. & Botha, C. J. Impact of pubertal imidacloprid exposure on the genital tract of Japanese quail (*Coturnix coturnix japonica*): Histomorphometric and ultrastructural study. *Tissue Cell* **96**, 102997 (2025). 40460538 10.1016/j.tice.2025.102997

[44] Mineau, P. & Kern, H. *Neonicotinoid insecticides: Failing to come to grips with a predictable environmental disaster* (American Bird Conservancy, 2023).

[45] Sonphule, A. M. et al. Effect of imidacloprid on growth performance and haemato-biochemical parameters in male Wistar rats. *Indian J. Vet. Pathol.* **43**(1), 38–42 (2019).

[46] Mohamed, W. H., Amein, K. A., Yahia, D., Sharkawy, A. & Mahmoud, A. S. Mutagenic effect of imidacloprid insecticide: The ameliorative effect of pre and post exposure to olive oil. *J. Food Biochem.* **44**(6), e13221 (2020). 32242959 10.1111/jfbc.13221

[47] Roy, C. L., Jankowski, M. D., Ponder, J. & Chen, D. Sublethal and lethal methods to detect recent imidacloprid exposure in birds with application to field studies. *Environ. Toxicol. Chem.* **39**(7), 1355–1366 (2020). 32274821 10.1002/etc.4721 PMC8164728

[48] Guzman, D. et al. High resolution, week-long, locomotion time series from Japanese quail in a home-box environment. *Sci. Data* **3**, 160036. 10.1038/sdata.2016.36 (2016). 27271772 PMC4896122

[49] Jung, H. & Dasen, J. S. Evolution of patterning systems and circuit elements for locomotion. *Dev. Cell* **32**, 408–422 (2015). 25710528 10.1016/j.devcel.2015.01.008 PMC4339819

[50] Brackenbury, J. Locomotion through use of the mouth brushes in the larva of *Culex pipiens* (Diptera: Culicidae). *Proc. Biol. Sci.* **268**, 101–106 (2001). 12123291 10.1098/rspb.2000.1336 PMC1087607

[51] Riedstra, B. & Roothius, T. G. Early feather pecking as a form of social exploration: The effects ofgroup stability on feather pecking and tonic immobility indomestic chicks. *Appl. Anim. Behav. Sci.* **77**, 127–138 (2002).

[52] Ali, H. M. et al. Imidacloprid effects on acetylcholinesterase and nicotinic acetylcholine receptor in *Apis mellifera*: Experimental and molecular modeling approaches. *Chemosphere* **352**, 141899 (2024). 10.1016/j.chemosphere.2024.141899 38579952

[53] Kamdem, M. M., Sithole, S. & Otomo, P. V. Effects of imidacloprid on the survival and biomarker responses of Eristalis tenax larvae (Diptera: Syrphidae): A comparative study between indoor and outdoor exposures. *J. Environ. Sci. Health B.* **59**, 333–340 (2024). 38660821 10.1080/03601234.2024.2343598

[54] Ijspeert, A. J. Biorobotics: Using robots to emulate and investigate agile locomotion. *Science* **346**, 196–203 (2014). 25301621 10.1126/science.1254486

[55] Gonzalez-Gallardo, A., Ramirez, F. J., Ortega, R. A. & Vidal, L. P. Biomarkers of nephrotoxicity in avian species. *Vet. Res. Commun.* **46**(2), 87–95 (2022).

[56] Johnson, R. J., Feehally, J. & Floege, J. *Comprehensive clinical nephrology* 6th edn. (Elsevier, 2019).

[57] Wankhede, V., Hedau, M., Ingole, R. & Hajare, S. Histopathological alterations induced by subacute imidacloprid toxicity in Japanese quails and its amelioration by Butea monosperma. *J. Pharmacogn. Phytochem.* **6**(3), 252–257 (2017).

[58] Akbas, D., Askin, A. & Çömelekoglu, Ü. Influence of neurotransmission in frog peripheral nerve by the neonicotinoid insecticide imidacloprid: An electrophysiological study. *Fresenius Environ Bull.* **23**, 1816–1823 (2014).

[59] Dutta, D., Ray, A., Bhattacharya, E., Ghosh, B. & Bahadur, M. Neurotoxic effects of imidacloprid on *Pethia conchonius* (Rosy Barb), a common freshwater fish of India. *Toxicol. Int*. (2024). 10.1080/01480545.2023.2222931 37326304

[60] Abdelhafez, H. E. D. H., Hammam, F. M., El-Dahshan, A. A., AboDalam, H. & Guo, J. Imidacloprid induces neurotoxicity in albino male rats by inhibiting acetylcholinesterase activity, altering antioxidant status, and primary DNA damage. *J. Toxicol.* 10.1155/2023/4267469 (2023). 37727350 PMC10506876

[61] Rahman, A. A. et al. Imidacloprid induced growth, hematological, neuro-behavior, anti-oxidant, economic, genetic, and histopathological alterations in *Clarias gariepinus*: Alleviative role of dietary *Hyphaene thebaica*. *Aquaculture* **564**, 739058 (2023).

[62] Hocking, P. M., Robertson, G. W. & Gentle, M. J. Effects of non-steroidal anti-inflammatory drugs on pain-related behaviour in a model of articular pain in the domestic fowl. *Res. Vet. Sci.* **78**, 69–75 (2005). 15500842 10.1016/j.rvsc.2004.05.005

[63] Houdelier, C. et al. Development of fearfulness in birds: Genetic factors modulate non-genetic maternal influences. *PLoS ONE* **6**(1), e14604. 10.1371/journal.pone.0014604.PMID:21298038;PMCID:PMC3029269 (2011). 21298038 PMC3029269

[64] Janner, D. E. et al. Oxidative stress and decreased dopamine levels induced by imidacloprid exposure cause behavioral changes in a neurodevelopmental disorder model in Drosophila melanogaster. *Neurotoxicology* **85**, 79–89 (2021). 34000340 10.1016/j.neuro.2021.05.006

[65] Zouaoui, S. & Rouabhi, R. Lysosomal disruption, mitochondrial impairment, histopathological and oxidative stress in rat’s nervous system after exposure to a neonicotinoid (imidacloprid). *Environ. Sci. Pollut. Res.* **31**(49), 59472–59489 (2024). 10.1007/s11356-024-35195-5 39356435

[66] Bathina, S. & Das, U. N. Brain-derived neurotrophic factor and its clinical implications. *Arch. Med. Sci.* **6**(6), 1164–1178 (2015). 10.5114/aoms.2015.56342 PMC4697050 26788077

[67] Janke, K. L., Cominski, T. P., Kuzhikandathil, E. V., Servatius, R. J. & Pang, K. C. Investigating the role of hippocampal BDNF in anxiety vulnerability using classical eyeblink conditioning. *Front. Psychiatr.* **6**, 106 (2015). 10.3389/fpsyt.2015.00106 PMC4513557 26257661

[68] Rodriguez-Carrillo, A. et al. Exposure to non-persistent pesticides, BDNF, and behavioral function in adolescent males: Exploring a novel effect biomarker approach. *Environ. Res.* **211**, 113115 (2022). 35292247 10.1016/j.envres.2022.113115

[69] Diz-Chaves, Y., Herrera-Perez, S., Gonzalez-Matias, L. C. & Mallo, F. Chapter fifteen_Effects of glucagon-like peptide 1 (GLP-1) analogs in the hippocampus. In *Vitamins and hormones* 118th edn (ed. Litwack, G.) 457–478 (Academic Press, 2022). 10.1016/bs.vh.2021.12.005 35180937

[70] Wojtowicz, S., Strosznajder, A. K., Jeżyna, M. & Strosznajder, J. B. The novel role of PPAR alpha in the brain: Promising target in therapy of Alzheimer’s disease and other neurodegenerative disorders. *Neurochem. Res.* **45**(5), 972–988 (2020). 32170673 10.1007/s11064-020-02993-5 PMC7162839

[71] Nierenberg, A. A. et al. Peroxisome proliferator-activated receptor gamma coactivator-1 alpha as a novel target for bipolar disorder and other neuropsychiatric disorders. *Biol. Psychiatry* **83**(9), 761–769 (2018). 29502862 10.1016/j.biopsych.2017.12.014

[72] Tabassum, S. et al. Resveratrol attenuates chronic unpredictable mild stressinduced alterations in the SIRT1/PGC1α/SIRT3 pathway and associated mitochondrial dysfunction in mice. *Mol. Neurobiol.* **60**(9), 5102–5116 (2023). 37256428 10.1007/s12035-023-03395-8

[73] Ford, K. A. & Casida, J. E. Chloropyridinyl neonicotinoid insecticides: Diverse molecular substituents contribute to facile metabolism in mice. *Chem. Res. Toxicol.* **19**(7), 944–951 (2006). 16841963 10.1021/tx0600696

[74] Schulz-Jander, D. A. & Casida, J. E. Imidacloprid insecticide metabolism: Human cytochrome P450 isozymes differ in selectivity for imidazolidine oxidation versus nitroimine reduction. *Toxicol. Lett.* **132**(1), 65–70 (2002). 12084621 10.1016/s0378-4274(02)00068-1

[75] Topal, A. et al. Neurotoxic responses in brain tissues of rainbow trout exposed to imidacloprid pesticide: Assessment of 8-hydroxy-2-deoxyguanosine activity, oxidative stress and acetylcholinesterase activity. *Chemosphere* **175**, 186–194 (2017). 28219821 10.1016/j.chemosphere.2017.02.047

[76] He, F. et al. New strategies for evaluating imidacloprid-induced biological consequences targeted to *Eisenia fetida* species and the corresponding mechanisms of its toxicity. *J. Environ. Manag.* **349**, 119456 (2023). 10.1016/j.jenvman.2023.119456 37897899

[77] Akinyemi, A. J. & Adewole, T. B-complex vitamins: An overview of their biological roles. *Niger. J. Biochem. Mol. Biol.* **36**(1), 5–14 (2021).

[78] Hanna, M., Jaqua, E., Nguyen, V. & Clay, J. B vitamins: Functions and uses in medicine. *Perm. J.* **26**(2), 89–97 (2022). 35933667 10.7812/TPP/21.204 PMC9662251

[79] Idriss, M. H., Khan, R., Patel, S. & Gomez, L. Role of B-vitamins in liver health: A clinical perspective. *Int. J. Clin. Nutr.* **43**(1), 15–25 (2025).

[80] Paleolog, J. et al. Imidacloprid markedly affects hemolymph proteolysis, biomarkers, DNA global methylation, and the cuticle proteolytic layer in western honeybees. *Apidologie* **51**(4), 620–630 (2020).

[81] Bashandy, S. A. E., Bashandy, M. A., El Zawahry, E. I., Abdel Naby, M. F. & Adly, F. Beta carotene and hesperidin antioxidants mitigate hepatotoxic effects of imidacloprid in male rats. *Al-Azhar Bull. Sci.* **28**, 15–27 (2017).

[82] Thornalley, P. J. & Rabbani, N. Vitamin B6 metabolism and its implications for human health. *Mol. Nutr. Food Res.* **55**(1), 137–152 (2011).

[83] Mastropasqua, F. et al. Neuroprotective effects of B-complex vitamins. *Neurotoxicology* **90**, 56–63 (2022).

[84] Genç, O., Yildiz, S., Kaya, E., Demir, A. & Aydin, M. B vitamins and neurological health. *J. Clin. Neurosci.* **112**, 40–49 (2024).

